# Time-course observation of tear film dynamics during VR headset use

**DOI:** 10.1038/s41598-025-16634-w

**Published:** 2025-09-26

**Authors:** Yoshiro Okazaki, Norihiko Yokoi

**Affiliations:** 1 Faculty of Human Sciences, Waseda University, 2-579-15 Mikajima, Tokorozawa-shi, Saitama 359-1192 Japan; 2 Department of Ophthalmology, Kyoto Prefectural University of Medicine, 465 Kajii-cho, Kawaramachi-Hirokoji, Kamigyo-ku, Kyoto, 602-8566 Japan

**Keywords:** Virtual reality headset, Dry eye disease, Tear film stability, Tear film lipid layer, Eye manifestations, Biomedical engineering, Health care

## Abstract

Dry eye disease is characterized by tear film instability, often linked to a reduced blink rate during prolonged visual display use. Although blink suppression during virtual reality (VR) headset use has been reported, its effect on tear film stability remains unclear. Accordingly, we developed a system using an ultra-compact camera for the time-course, noninvasive observation of tear film dynamics. Fourteen healthy participants played a 30-min VR game while tear film kinetics were analyzed. As gameplay progressed, a significant increase in the interference grade of the tear film lipid layer was observed (*p* < 0.05), along with elevated corneal and upper eyelid surface temperatures (*p* < 0.05). The increased interference grade suggests thickening of the lipid layer, possibly attributable to a periocular temperature elevation which may result in facilitation of the incorporation of polar lipids into the nonpolar lipid layer. These findings suggest that VR headset use may increase lipid layer thickness, potentially improving tear film stability under these conditions.

## Introduction

Dry eye disease is an ocular condition characterized by tear film instability, which leads to ocular discomfort and visual impairment. More than one billion people worldwide are estimated to be affected by this condition, with the associated economic burden reaching approximately USD 3.86 billion in the United States alone^1^. One of the key etiological factors is prolonged visual display terminal use, which reduces blink rate and facilitate aqueous tear evaporation from the ocular surface. This process destabilizes the tear film, resulting in the tear film breakup^2^. Although voluntary blinking has been recommended as a preventive measure for dry eye, it is not widely practiced in daily life. Accurate evaluation of the tear film is challenging due to its small volume and transparent nature. In the clinical setting, invasive techniques such as the instillation of fluorescein dyes followed by slit-lamp biomicroscopy are commonly used. Alternatively, noninvasive methods based on observation of the interference patterns of the tear film lipid layer have been developed^3^. Several commercial interferometric devices are available, but they are large-scale medical instruments intended for use in clinical environments, making tear film evaluation impractical in non-clinical settings.

In recent years, the virtual reality (VR) gaming market has grown rapidly: its global value reached USD 18.3 billion in 2020 and is projected to expand at an annual growth rate of approximately 28% through 2026^4^. A key feature of VR gaming is the immersive experience it offers, allowing users to feel as though they are physically present in a virtual environment. Players wear VR headsets that provide fully immersive 3D visuals, creating a strong sense of realism by disconnecting them from the physical world. However, these headsets place considerable strain on the eyes, as users must continuously focus on dynamic images from a screen at a very short viewing distance. Although appropriate usage guidelines are recommended, few studies have examined the ocular safety of VR headsets, particularly their effects on the tear film.

Previous studies have reported that blink frequency decreases and ocular discomfort increases during VR headset use^5,6^. In addition, while some studies have shown that tear film stability improves after VR use^7^, others have found no significant change^8^, and a consensus has not yet been established. Importantly, these studies have primarily focused on comparisons made before and after VR headset use. However, to accurately evaluate the impact of wearing a VR headset on the tear film, it is essential to capture the dynamic behavior of the tear film during headset use.

Therefore, the aim of this study was to clarify the effects of VR headset wear on tear film stability through time-course, noninvasive observation of tear film dynamics using interference patterns of the lipid layer.

## Subjects and methods

### Subjects

The study involved the right eyes of 14 healthy participants (9 men and 5 women; mean age, 29.8 ± 13.6 years), all examined without visual correction (unaided eyes). Individuals with a history of ocular surgery or dry eye disease were excluded. Four contact lens wearers were included in the study because they represent a common subgroup in the general population and may exhibit different tear film dynamics due to chronic ocular surface stress. Participants who normally wore glasses were instructed to remove them 10 min before the experiment; contact lens users removed their lenses 30 min beforehand.

### Principle of interference-based measurement of the tear film lipid layer

When white light is projected on a thin film with a refractive index from that of air, a phase shift occurs in the reflected light due to the difference of the optical path length between reflections at the film interfaces. Specifically, constructive interference (bright fringes) occurs when the optical path difference is an integer multiple of the wavelength, whereas destructive interference (dark fringes) occurs when it is a half-integer multiple. Let *n* be the refractive index, $\lambda$ the wavelength of light, *d* the thickness of the lipid layer, $\theta$ the angle of reflection at the interface, and *m* an integer. Under these conditions, the equation for constructive interference is given by (1):

1 $$\begin{aligned} 2d \cos \theta = \left( m + \frac{1}{2}\right) \frac{\lambda }{n} \end{aligned}$$

By utilizing this principle of optical interference, the tear film lipid layer can be noninvasively visualized. This enables both qualitative grading and dynamic analysis of the lipid layer based on its interference patterns^9–11^.

### Experimental system

**Fig. 1:**
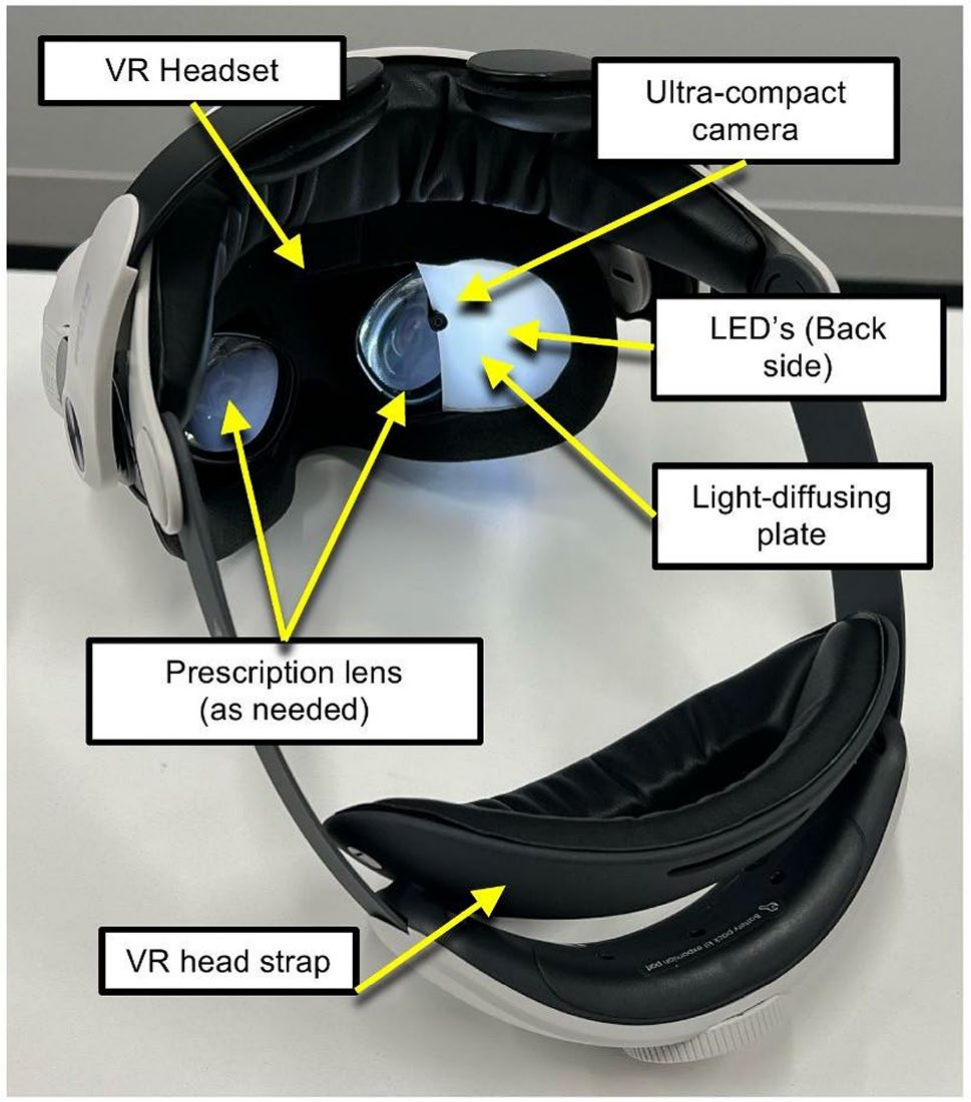
External view of the tear film observation system integrated into a VR headset.

The tear film observation system mounted on a VR headset is shown in Fig. 1. The VR headset used was the Meta Quest 2 (Meta Inc.). A light-diffusing plate (SW-12, Nikon Corp.) was installed inside the headset, covering approximately one-third of the right lens area. The light-diffusing plate was thermally molded into a non-spherical concave shape to satisfy multiple design constraints and ensure uniform illumination across the entire corneal surface. Specifically, the plate was mounted inside the VR headset’s foam cushion to avoid contact with the eyes or eyelashes. A flat plate would pose a risk of such contact, whereas excessive curvature could interfere with the VR lenses. LEDs (LED3HAUDL05M, JTT Corp.) were arranged behind the diffuser such that the entire surface of the plate emitted light.

An ultra-compact camera (XT-ZL31105-158, Misumi Electronics) was positioned at the left edge of the diffuser, near the center of the right lens, enabling a wide field of view for corneal surface imaging. The camera had a field of view of 158$^\circ$, a focal length of 20 mm, and a depth of field ranging from 9 to 24 mm. A sheet-type heat sink was attached to both the camera and the LED driver board to dissipate heat externally from the enclosure.

To maintain an adequate distance between the eye and the camera, a standard eyeglass spacer was inserted. A VR head strap (M2 Plus, BOBOVR) was used to stabilize the device position during gameplay. Camera images were transmitted via USB cable to a laptop computer for data acquisition. Prescription lens inserts for VR headsets (VirtuClear Inc.) were used as needed.

### Experimental procedure

The experiment was conducted in a controlled laboratory setting at Waseda University (temperature, 24.4 ± 2.0 $^\circ$C; humidity, 37.4 ± 9.0%). Participants wore the VR headset for a continuous session of 30 min, played a VR game, *Tetris*®*Effect: Connected* (Enhance Inc.), during which tear film dynamics were recorded. Due to the considerable eye movement associated with gameplay, it was difficult to obtain comparable measurements over time under consistent conditions. Therefore, tear film measurements were performed at baseline (0 min) and subsequently at 5-min intervals: 5, 10, 15, 20, 25, and 30 min. For each measurement, gameplay was briefly paused and was resumed immediately upon completion of the imaging.

In accordance with previously reported methods^12–14^, participants were asked to gently close their eyes and then maintain eye opening for as long as possible, up to a maximum 10 s or until discomfort occurred. This procedure was intended to ensure consistent initial conditions across time points and was repeated three times per time point, with the average of the three measurements taken as the representative value.

Before and after the VR session, participants completed a questionnaire regarding dry eye symptoms. In addition, the temperatures of three sites—the corneal surface of the right eye, the upper eyelid surface of the right eye during eye closure, and the inner surface of the right lens of the VR headset—were measured using a thermographic camera (FLIR One Pro, FLIR Systems). These thermal images were captured within 10 s after removal of the headset to minimize heat dissipation, with the camera positioned approximately 30 cm from the target. The air temperature inside the VR headset was not monitored during the experiment.

### Analysis protocol

The tear film lipid layer, primarily comprising lipids secreted from the meibomian glands, is thought to play a critical role in preventing aqueous tear evaporation. An increase in the thickness of the tear film lipid layer has been reported to produce multicolored interference fringes due to optical interference phenomena^3,9,15^.

**Fig. 2:**
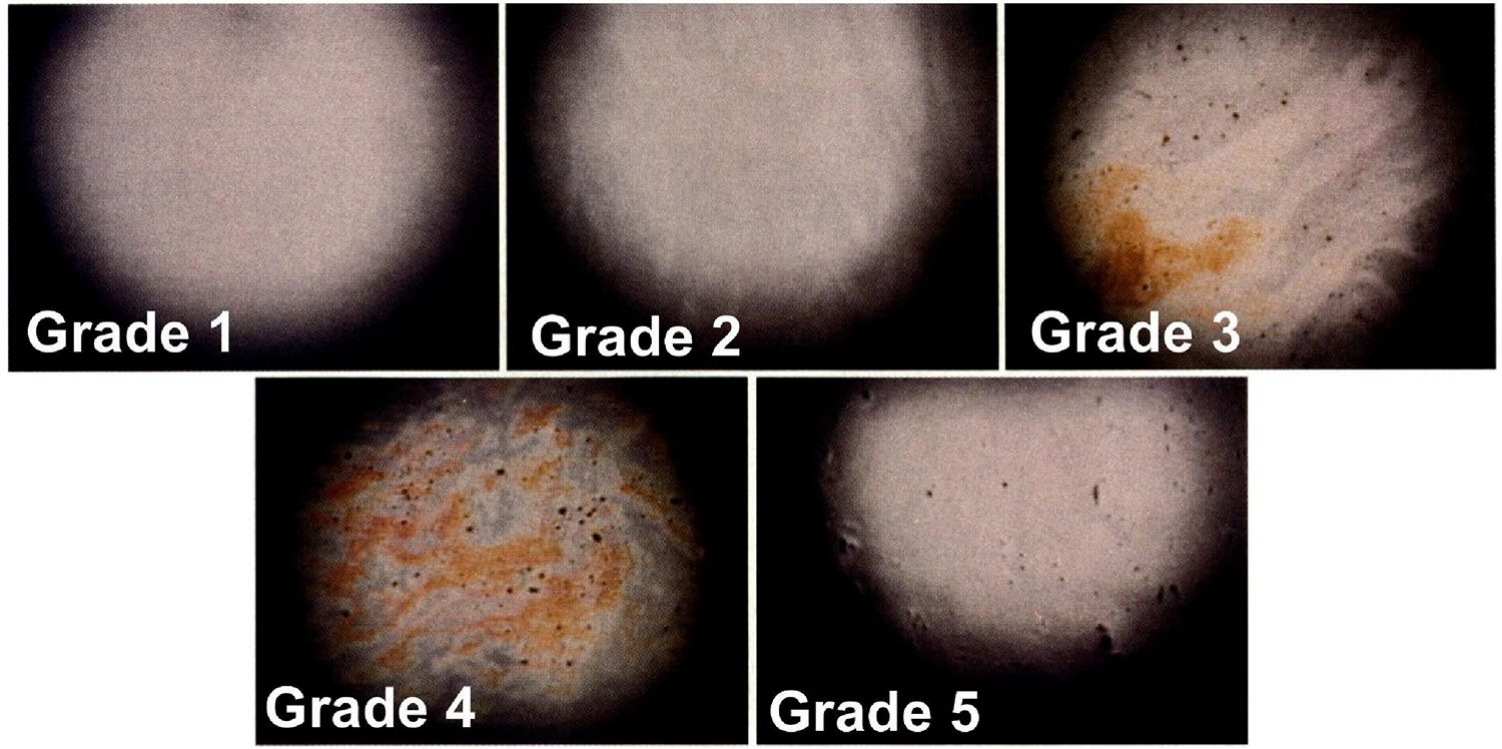
Grading classification of tear film lipid layer interference patterns (adapted from reference^3^).

**Fig. 3:**
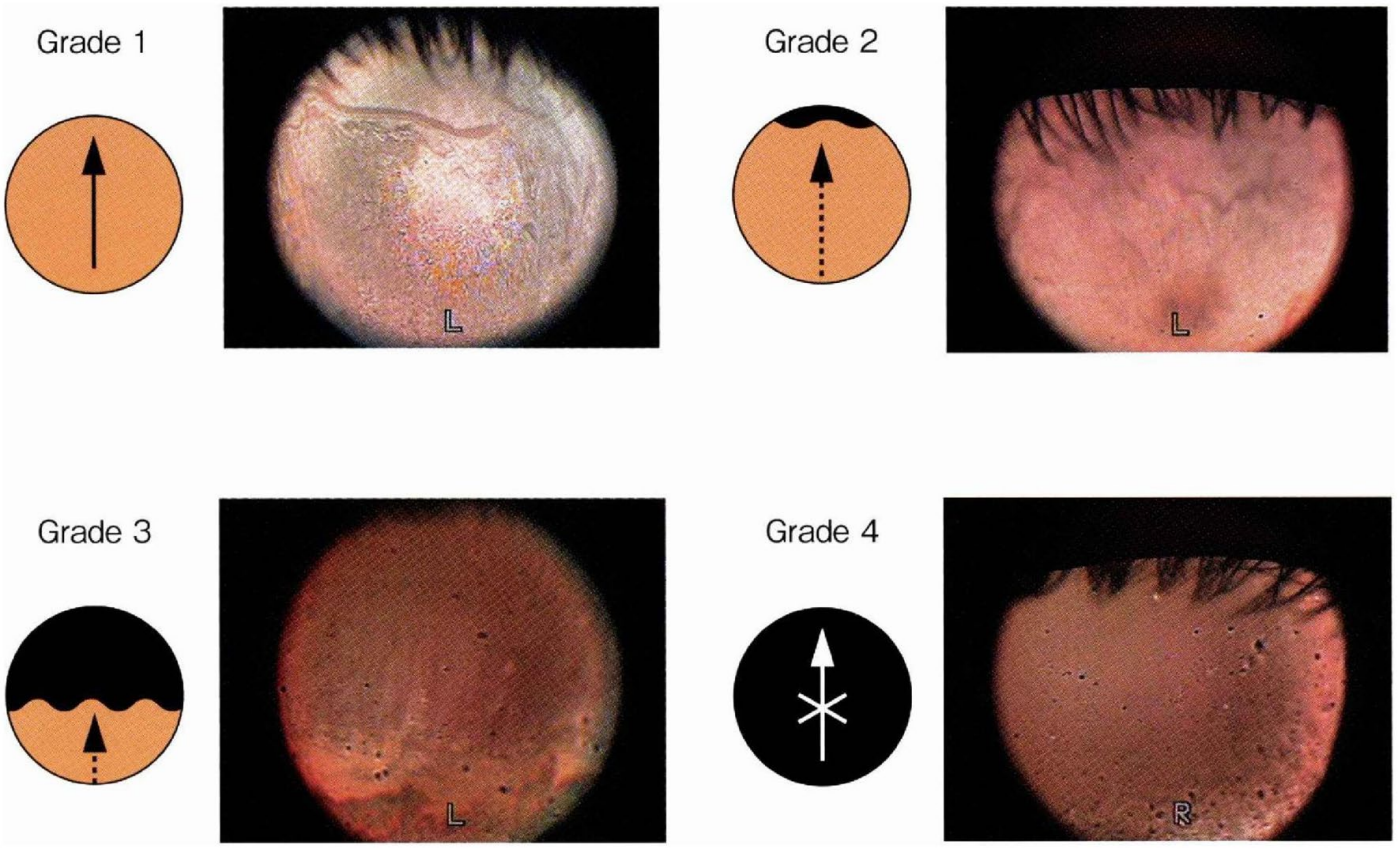
Grading classification of lipid layer spreading on the cornea after eye opening (adapted from reference^10^).

In this study, both the interference patterns and the following five parameters related to the tear film were analyzed, based on previously reported methods^3,14–17^: (1) Interference grade of the tear film lipid layer (Fig. 2), (2) Upward spread grade of the lipid layer on the cornea after eye opening (Fig. 3), (3) Lipid spread time (LST): the interval from full eye opening to the first frame at which the lipid layer showed no further upward motion, identified by frame‑by‑frame analysis of interferometric images using image analysis software,^17^, (4) Maximum blink interval (MBI): the maximum duration the eyes can remain open without blinking, measured after a blink by instructing participants to keep their eyes open for as long as possible until the next spontaneous blink occurred,^14^, (5) Tear meniscus height (TMH): the height of the lower tear meniscus, used as an indicator of tear volume.

In addition, subjective dry eye symptoms were assessed using a visual analog scale (VAS) for the following 12 items: eye dryness, blurred vision, photophobia, eye fatigue, heavy eyelids, eye pain, foreign body sensation, difficulty in opening the eyes, eye redness, tearing, eye itchiness, and eye discharge.

The interference grade (1) was classified in accordance with previous studies^3,15^. Grade 1 was defined as a uniform gray interference appearance with homogenous lipid distribution; Grade 2 indicated a gray interference color with visible fringes; Grade 3 was assigned when interference colors other than gray were observed; and Grade 4 was defined by the presence of multicolored interference fringes.

For the lipid spread grade (2), the extent of upward lipid layer spread on the cornea following eye opening was evaluated. Grade 1 was defined as smooth and complete spreading across the entire corneal surface; Grade 2 was assigned when the lipid layer slowly spread over more than half of the cornea; Grade 3 corresponded to slow spreading over less than half of the cornea; and Grade 4 indicated no apparent spreading. The measurement site for TMH (6) was defined as the most central part of the lower eyelid margin, as visualized in the reflected image of the tear meniscus. The height was estimated in reference to the diameter of the black circular shadow cast on the cornea by the compact camera, using the standard anatomical size of the cornea as a scale.

Statistical analyses were performed using RStudio (R version 4.3.1). For each tear film parameter, the Friedman test and Wilcoxon signed-rank test were applied, with a two-sided significance level set at 5 percentage. Paired t-tests were used to compare temperature and VAS data before and after the experiment. Image analysis of the tear film was conducted using ImageJ software (version 1.54fi). All methods were performed in accordance with the Declaration of Helsinki and the relevant institutional guidelines and regulations. This study was approved by the Ethics Committee of Waseda University (approval number: 2023-110). Written informed consent was obtained from all subjects after the nature and possible consequences of the study had been fully explained.

## Results

**Fig. 4:**
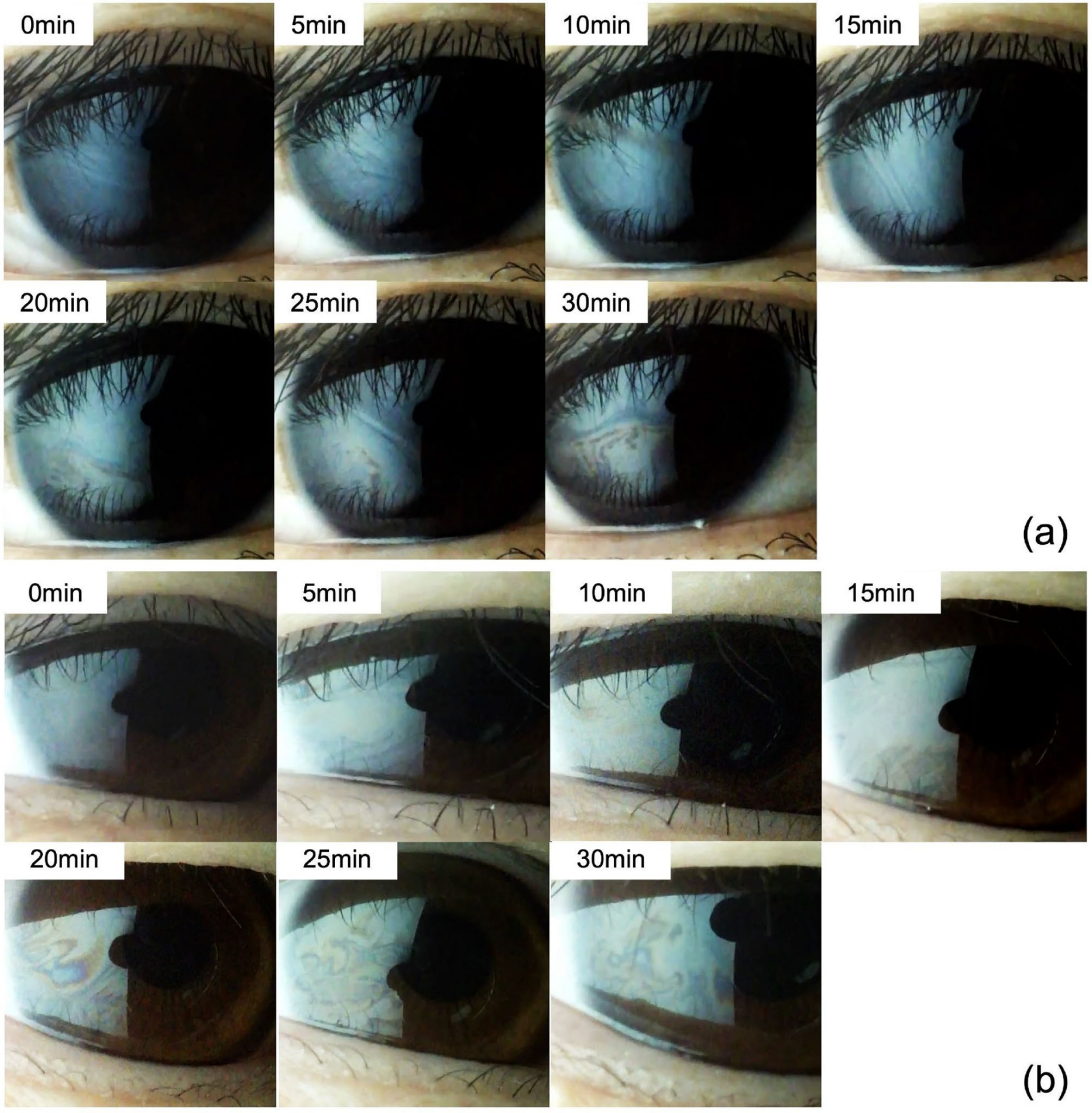
Example of a tear film image captured during VR headset use. (**a**) and (**b**) show images obtained from two different participants. (contrast enhanced by 15% to improve interference pattern visibility).

Tear film dynamics during VR headset use were successfully observed through time-course measurements. A representative example of the time-series changes in the tear film lipid layer images is shown in Fig. 4, confirming that the lipid layer could be clearly visualized during VR headset use.

**Fig. 5:**
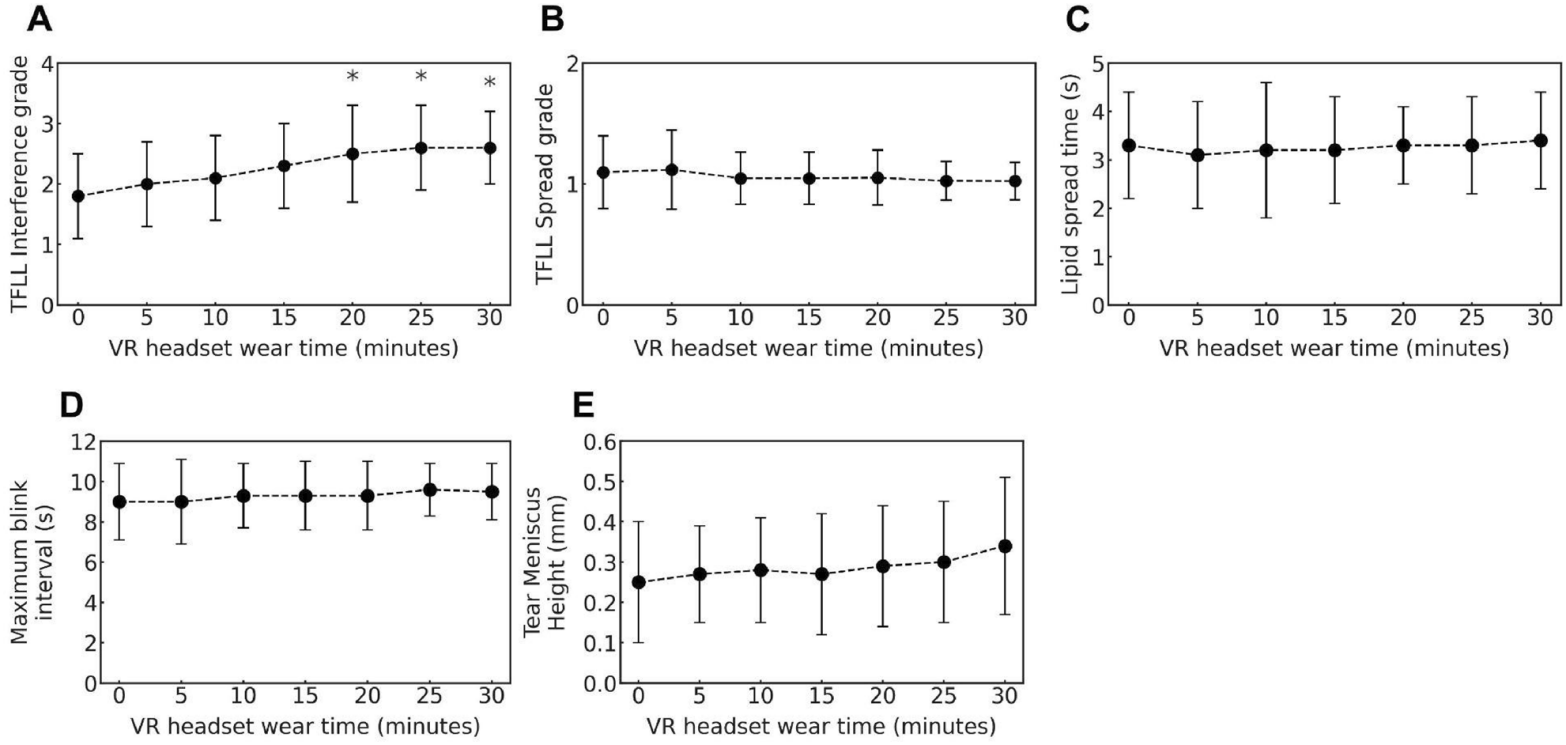
Time-dependent changes in the parameters related to the tear film; (**A**) interference grade of the tear film lipid layer, (**B**) spread grade of the tear film lipid layer, (**C**) lipid spread time (LST), (**D**) maximum blink interval (MBI), (**E**) tear meniscus height (TMH).

Figure 5A illustrates the changes in the interference grade of the tear film lipid layer during VR gameplay. The mean grade at the start of the measurement was 1.8 ± 0.7. Over time, the interference colors gradually became more vivid, and a significant increase was observed after 20 min of headset use (*p* < 0.05). The grades at 20, 25, and 30 min were 2.5 ± 0.8, 2.6 ± 0.7, and 2.6 ± 0.6, respectively. The median difference in interference grade between the beginning of the session and 20 min was + 1.5 while that between the beginning and 30 min was + 1.0.

In addition to interference grade, other tear film stability-related parameters—including lipid spread grade, LST, MBI, and TMH—were analyzed over time. The results are shown in Fig. 5B through E. No significant differences were observed across time points for any of these parameters (*p* > 0.05). The mean values over the 30-min period were as follows: spread grade, 1.1 ± 0.2; LST, 3.3 ± 1.1 s; MBI, 9.3 ± 1.7 s; and TMH, 0.29 ± 0.15 mm.

**Fig. 6:**
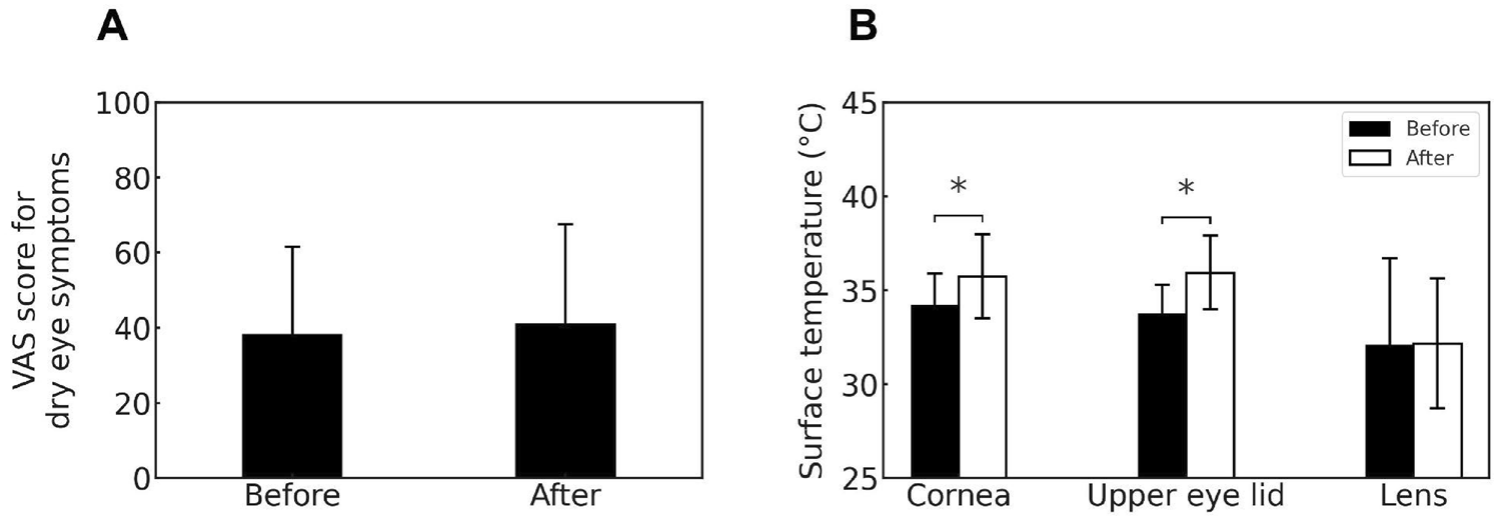
Subjective dry eye symptoms (**A**) and surface temperatures of ocular and device-related regions (**B**) before and after the experiment.

The total VAS scores for subjective dry eye symptoms before and after the experiment are shown in Fig. 6A . The mean VAS score was 38.1 ± 23.3 before the experiment and 40.8 ± 26.8 after the experiment, with no significant difference being observed (*p* > 0.05). The surface temperature of the right cornea increased significantly from 34.2 ± 1.7 $^\circ$C before the experiment to 35.7 ± 2.2 $^\circ$C afterward (*p*<0.05) (Fig. 6B , left). The upper eyelid surface temperature of the right eye also increased significantly, from 33.8 ± 1.5 to 35.9 ± 1.9 $^\circ$C (*p* < 0.05) (Fig. 6B, middle). In contrast, no significant difference was observed in the surface temperature of the right lens inside the VR headset (*p* > 0.05) (Fig. 6B, right).

## Discussion

By incorporating an ultra-compact camera and illumination unit inside the VR headset, time-course observation of tear film dynamics during VR gameplay was achieved. Over time, the interference colors of the tear film lipid layer gradually shifted from gray to multicolor, and a significant increase in interference grade was observed after 20 min. This finding indicates that the lipid layer thickened as a result of VR headset wear. This result is consistent with a previous study by Turnbull et al.^7^ that reported that the thickness of the tear film lipid layer increased following VR headset wear, leading to enhanced tear film stability. They suggested that this thickening may be attributable to stimulated lipid secretion from the meibomian glands. However, if lipid secretion were continuously stimulated, the interference grade of the tear film lipid layer would be expected to continue increasing after 20 min. In the present study, however, no significant change was observed beyond 20 min, and the grade appeared to stabilize after reaching a certain level.

Georgiev et al.^18^ hypothesized that elevated temperature increases the fluidity of nonpolar lipids in the tear film lipid layer, making them behave more like a “lipophilic fluid”. This change could alter the distribution of polar lipids by increasing their partitioning into the oily layer, resulting in thicker meibum films at higher temperatures. In the present study, the average surface temperatures of the cornea and upper eyelid increased by 1.5 and 2.1 $^\circ$C, respectively, after the experiment compared to baseline values. This temperature elevation was accompanied by an increase in the interference grade of the tear film lipid layer. These findings are consistent with the hypothesis proposed by Georgiev et al. Consistent with the findings of Georgiev et al., an increase in periocular temperature inside the goggles enhances the fluidity of the tear film lipid layer. At 34 $^\circ$C, the non-polar lipid layer of the tear film can be regarded as a lipophilic fluid. This increased fluidity promotes the reorganization of the tear film lipid layer by facilitating the incorporation of polar lipids into the non-polar lipid layer. As a result, the thickness of the tear film lipid layer is considered to increase. The absence of further changes in lipid layer thickness observed after 20 min in our study suggests that the temperature elevation inside the goggles led the tear film lipid layer to reach a new structural steady state, beyond which no significant structural changes occurred.

The absence of change in parameters related to tear film lipid layer dynamics, such as spread time and spread grade, may be due to the unchanged thickness of the aqueous sublayer located directly beneath the lipid layer, which is linearly correlated with TMH^11^. In other words, because TMH did not change, no significant changes were observed in lipid layer parameters other than interference grade.

An increase in interference grade indicates an increase in the thickness of the tear film lipid layer, and there are reports that when such thickening is induced by elevated environmental temperature, it may be accompanied by improved tear film stability. However, excessive thickening of the tear film lipid layer may also contain abnormal lipids as part of its composition, and whether it is accompanied by improved tear film stability requires further verification. Interestingly, no significant change in dry eye symptoms was reported despite the observed increase in lipid layer thickness. This dissociation highlights the well-known disconnect between clinical signs and subjective symptoms in ocular surface disorders.

In a subgroup analysis, we compared the time course of interference grade changes between contact lens wearers and non-contact lens wearers. In non-contact lens wearers, the interference grade began to increase after 15 min of VR use, while in contact lens wearers, a similar increase was observed only after 25 min (*p* < 0.05). This temporal difference suggests that prior contact lens wear may influence the dynamics of lipid layer response to VR exposure.

Another factor that should be considered when interpreting the experimental results is the shielding effect of the VR headset, which covers the eyes and protects the ocular surface from ambient airflow, such as that of air conditioning. This protection has been suggested to reduce tear evaporation and promote tear film stability^7^. Kimball et al. reported that sealed goggles can significantly suppress aqueous thinning^19^.In addition, Li et al. demonstrated that ocular surface cooling is directly associated with tear evaporation^20^. In the present system, small cable ports for the camera and other components were installed on the sides of the face guard. Therefore, while the sealing effect may have been lower compared with a standard VR headset, it is considered that the shielding effect still contributed to reducing tear evaporation.

There are several limitations to this study. First, although the present study focused on healthy individuals, it remains unclear whether the lipid layer responses to VR headset wear observed here would occur similarly in individuals with dry eye disease or meibomian gland dysfunction. Because tear film lipid layer behavior can differ under pathological conditions, further studies involving clinical populations are warranted to assess the generalizability of these findings. Second, the 30-min washout period set for contact lens wearers in this study was shorter than that used in some previous studies. However, there are reports indicating that, in CL wearers, changes in tear reservoir volume and tear film stability return to baseline within 15 min after lens removal. This should be taken into account when interpreting the findings. Third, the absence of a control group, such as participants wearing a non-VR headset or no headset at all, is another limitation. Future studies should incorporate appropriate controls to eliminate potential confounding factors, including ambient temperature and insulation effects. Finally, it is necessary to directly measure the air temperature and humidity inside the headset, as these factors may influence tear evaporation.

## Conclusion

This study developed a system for time-course observation of tear film dynamics using an ultra-compact camera embedded in a VR headset. In a 30-min VR gaming task with healthy participants, the interference grade of the tear film lipid layer significantly increased over time, in addition to the surface temperatures of the cornea and upper eyelid. These findings suggest that VR headset use may enhance tear film stability by promoting lipid layer thickening through a localized periocular temperature rise.

## Data Availability

The data presented in this study are available upon reasonable request from the corresponding author.
